# Supplementary material for: Participatory peer research exploring the experience of learning during Covid-19 for allied health and healthcare science students
Source: PLoS One. 2022 Oct 27;17(10):e0276180. doi: 10.1371/journal.pone.0276180 (PMC9612578; doi:10.1371/journal.pone.0276180)
Supplement: Attachment — Submitted filename: Response to reviewers.docx [file pone.0276180.s001.docx]

Dear Editor,

Thank you for the opportunity to enhance and revise this manuscript. The following letter outlines the authors response to reviewer’s comments.

We believe the manuscript is formatted according to style requirements

Funding Statement:

This work was supported by the Council for Allied Health Professionals Research (CAPHR) and The Centre for Health and Rehabilitation Technologies (CHArT). 
Both funds provided small amounts of money directly to the project team, CAPHR provided funds for interview transcription and CHArT provided funds for two peer researchers to engage in analysis. 
CAPHR: <https://cahpr.csp.org.uk>
CHArT:<https://www.ulster.ac.uk/research/topic/nursing-and-health/chart>

Any funding related information was removed from the manuscript.

Data availability

Data cannot be shared publicly because of ethical restrictions on sharing sensitive data. Data are available from the Ulster University Institutional Data Access (pure-support@ulster.ac.uk) for researchers who meet the criteria for access to confidential data. This can be obtained through this link: https://pure.ulster.ac.uk/en/datasets/qualitative-participatory-peer-research-exploring-the-educational

| Reviewer 1 comment | Response to reviewer |
| --- | --- |
| The study tried to use the DEPICT Framework to Analysis the interview result is good, yet, suggest authors address more related interview guidelines, coding book, coding analysis method, for instance, how to get the Themes and sub-themes by table 2, the section unclear. | Thank you for highlighting that the data analysis section was unclear. This section has been revised to take into consideration the core principles of thematic analysis that was used within the DEPICT framework. It is great to have to opportunity to expand on this and make it clearer.  For transparency, this approach was guided by the work of Braun and Clark (multiple publications although the reference below would be helpful to refer to for clarity).  I am not completely sure what this reviewer is asking for as I believe we have experience of different qualitative approaches. I have attempted to generate more clarity in the development of codes into themes through the Mural images (Figure 1 and 2).  Braun V, Clarke V. Can I use TA? Should I use TA? Should I not use TA? Comparing reflexive thematic analysis and other pattern-based qualitative analytic approaches. Couns Psychother Res. 2021;21(1):37–47. |
| On page 7, "…we aimed to recruit 10 participants to each session….” But the Results section shows that “Twenty-three participants participated in six focus groups ranging from 2 to 5 participants….” So what are the actual samples? | This statement has been removed for clarity. The sample is 23 participants. |
| -On page 7, Although the authors presented the “Table 1: Data Analysis using the DEPICT Framework,” what’s the detailed codebook related Themes and sub-themes also unclear, so how to mentation the result is contributed. | To provide clarity around the themes and codes Figure 1 is a screenshot of early codes in a subset of the data. Figure 2 presents the final themes and sub-themes through the Mural platform. |
| -On page 7, “The focus groups were conducted using the platform Blackboard Collaborate Ultra” the platform should address how to recruit different professional field participants to join the platform. | I am unsure what this reviewer is asking for. If I have not addressed it properly, I am happy to with more clarity.  The following was added: ‘The focus groups were conducted using Blackboard Collaborate Ultra, that is part of the University Blackboard Learn platform. All students regularly use this platform to participate in teaching and learning.’ |
| - Data analysis section, “The data analysis was undertaken in two separate parts with two different teams. All transcripts were coded by one person and reviewed by two people.” For more contributions, please add Coder reliability and validity. | Further clarity on the data analysis was expanded upon and approaches used to support the trustworthiness and credibility of the data was described. Rigor was achieved through reflexive journaling, peer researcher review, regular meetings, and deep discussion. Participatory data analysis uses the subjective interpretation of the data by people with lived experience. Through discussion and reflection of this experience richer data collection, discussion and interpretation could but achieved.  I am not sure if the reviewer is looking for inter-rater reliability. This was not in line with our approach. This does not support the rich multiple coder approach taken in the study. See this paper for supporting evidence.  Sweeney, Angela, Kathryn E. Greenwood, Sally Williams, Til Wykes, and Diana S. Rose. "Hearing the voices of service user researchers in collaborative qualitative data analysis: the case for multiple coding." *Health Expectations* 16, no. 4 (2013): e89-e99. |
| -up to the topic is “Qualitative participatory peer research exploring the educational impact of Covid-19 on Allied Health and Healthcare Science Students” however the study related to educational activities on Allied Health and Healthcare Science Students is unclear, so the topic may need modification. | The manuscript refers to the process (peer researchers) and outcome (experience of covid). It is difficult to unpick the two as peer researchers explored the impact Covid had on their peers, and they are all allied health and healthcare science students. I hope that the revisions within the manuscript help to clarify and mesh the process and outcome into a seamless thread throughout the paper. |

| Reviewer 2 comment |  |
| --- | --- |
| 1.Title is too long and everything is included in the title: Qualitative participatory peer research…… I think, Participatory peer research exploring the educational….. is appropriate. | Thank you for this recommendation. The word Qualitative has been removed from the title in line with this recommendation. |
| 2.In the Abstract, some claiming sentences are written at the beginning of the abstract without any evidence and appropriate support. How could you say the global pandemic had an impact on teaching and learning activities in health and health care students? | This sentence was changed to say that the teaching and learning has altered for students, as all campus-based teaching and learning activities did during the pandemic. As it is not common practice to use citations in an abstract, I hope this is an appropriate change in line with your recommendation. |
| 3.On page 2, line 17, it is written that the knowledge generated from this project was co-production using an online format, including the dissemination. What does it mean? | In line with the revision made to the conclusion this sentence was removed. |
| 4.In the introduction section, different previously done researches are mentioned. But, a single paragraph gives multiple ideas, which is not good in academic writing. In the introduction, a discussion about allied health students is missing. This could be new for non-health sector readers | The introduction section has been revised and I hope this makes it easier to read with single ideas presented per paragraph. An introduction to allied health professions and healthcare science students was also added. |
| 5.In the methods section, it is mentioned that participatory action research (PAR) was adopted in the research. But, this study does not carry any essence of PAR. Participatory action research requires participants to build and keep evidential records of practice, theory, and reflection and to provide a reasoned justification to others for their work. Doing this requires authentic participation and is collaborative, establishing self-critical, non-hierarchical communities and partnerships. It is also a recursive and systematic process of learning, with planning, action, analysis, and reflection leading to further planning, action, analysis, and reflection. Only, the peer research would be appropriate but it is not action research. The methods section also misses why peer research was used.  6.Ethical issues in doing the qualitative peer research are missing. How these issues were handled? | I think the reviewer highlights an important point. Thank you for this thoughtful reflection. The research was participatory however doesn’t quite meet the threshold for ‘action’ as you rightly state. It was not an iterative approach in the evidence gathering although it was iterative in skill development. Participatory approach is presented and explored. An extensive description of the peer researcher approach and underpinning rational was added to the methods section. Emphasis was also placed on the ethical challenges. |
| 7.Conclusion of the study does not hold the essence of the overall result and discussion. | A review of the manuscript was undertaken, and a revised conclusion is now presented. |
| At last, this paper could be made publishable after the rigorous work in the methods section. Does it actually a PAR? Does it complete all the processes of PAR? | The revision focused on creating a robust methods section, highlighting participatory research and the partnership approach taken within the study. The revision also enhanced the data analysis approach and rigor adopted. |

Thank you for the opportunity to enhance this manuscript. Please do not hesitate to contact me for further reflection or recommendations.

Kind regards

Jean
