# Supplementary material for: Participatory peer research exploring the experience of learning during Covid-19 for allied health and healthcare science students
Source: PLoS One. 2022 Oct 27;17(10):e0276180. doi: 10.1371/journal.pone.0276180 (PMC9612578; doi:10.1371/journal.pone.0276180)
Supplement: Attachment — Submitted filename: Response to Reviewers.docx [file pone.0276180.s002.docx]

Dear Editor,

Thank you for the opportunity to enhance and revise this manuscript. The following letter outlines the authors response to reviewer’s comments.

| Reviewer 1 comment | Response to reviewer |
| --- | --- |
| 1. Due to the title being “Participatory pee 1 r research exploring the educational impact of Covid-19 on Allied Health and Healthcare Science Students” the platform should address how to recruit different professional field participants to join the platform. Please clarify how to ensure different professional field participants’ baseline, although the authors stated that “All students regularly use this platform to participate in teaching and learning.’” | The line in relation to this question was identified on Pg. 9 line 12-13. I believe this query is around the use of the Blackboard Collaborate Ultra platform. Clarifying text has been added line 11 and line 15 to highlight the online meeting room could be accessed by any participant with a meeting link. The entire sample, which is any registered student from the different professional’s fields, would have equal access to participation given that they use Blackboard for their teaching and learning at the University. In addition, the weblink gives access to the meeting room. The participants were recruited from programmes registered within the school of health sciences and therefore had equal opportunity to participate in a focus group. |
| 2. Data analysis section, “The data analysis was undertaken in two separate parts with two different teams. All transcripts were coded by one person and reviewed by two people.” In addition, on page 11, the study presented that “…through data collection and analysis supported the rigorous analytic approach with multiple partners…” please add multiple partners’(coder) reliability and validity. | Participatory data analysis uses the subjective interpretation of the data by people with lived experience. Through discussion and reflection of this experience richer data collection, discussion and interpretation could be achieved.  Coder reliability and validity was not used as part of this approach and therefore cannot be provided in this manuscript. Coding reliability approaches measure the level of agreement between coders (Guest et al., 2012). The participatory approach uses the DEPICT framework to harness the meaning and understanding of the data by multiple (peer) researchers to create depth not to measure the level of agreement. Pg. 10 line 10-17 seeks to address the reason for the coding book as a scaffolding to support collaborative data analysis. The sentence Pg. 11 line 17-19 stated *‘the* ***consistency of one researcher*** *(JDL) through data collection and analysis supported the rigorous analytic approach with multiple partners.*’ A singular researcher enabled consistency and ensured shared meaning across the two teams analysing the data. |
| 3. up to the topic is “Qualitative participatory peer research exploring the educational impact of Covid-19 on Allied Health and Healthcare Science Students” however, the study related to educational activities on students is unclear, so suggest authors address the study’s educational activities, such as the online class via research activities process, detailed research content, and task. They are unclear. Thank you. | Thank you for this comment. I have changed the title slightly as a response. This study aims to capture the experiences students had as they navigated the pandemic on their journey to becoming allied health professionals and healthcare scientists. The ‘educational impact’ was misleading as a qualitative piece of work. |

| Reviewer 2 comment | Response to reviewer |
| --- | --- |
| The authors have revised their manuscript as recommended in the first draft. However, I have a remark regarding the role of peer researchers in the process of study. The role of peer researchers is seen only in data collection procedures then how could you say they were peer researchers? The rest of all is fine and the manuscript can be published. | Thank you for the recommendation to publish this manuscript. Just to highlight the peer researcher role was throughout the lifespan of the project. Pg. 6 line 4-5 sets this out briefly. In addition to collecting the data, peer researchers reviewed the research protocol and ethical documentation, co-designed the topic guide, analysed the data and have supported various different dissemination strands in the project. |

Thank you for the opportunity to enhance this manuscript. Please do not hesitate to contact me for further reflection or recommendations.

Kind regards

Jean
